# Medical Periodicals

**Published:** 1855-09

**Authors:** 


*Medical Periodicals in the United States.*—" During the last seven years many changes have taken place in our periodical medical literature. There were in 1847, in the United States and the adjacent British Provinces, *eighteen* periodicals devoted to the interests of medicine and its collateral branches. Ours was the nineteenth enterprize of the kind. Of these nineteen, *five* have ceased to exist. Since the Reporter was commenced, *thirty-three* new periodicals have been commenced—nearly five a year. Of these, *fourteen*, or two a year, have been discontinued. During the last seven years, therefore, as many Journals have been discontinued as were in existence at the commencement of that period, and the number now in existence equals those that have been commenced in that time. There are also four re-prints, of foreign medical works, making in all *thirty-seven* periodicals now existing in this country, devoted to the interests of medicine !"—*N. J. Med. Reporter.*

